# An unbalanced time-perspective profile in cardiac surgery patients as a risk factor for depression

**DOI:** 10.1192/j.eurpsy.2021.2007

**Published:** 2021-08-13

**Authors:** O. Nikolaeva, E. Nikolaev, S. Petunova, N. Grigorieva, E. Lazareva, D. Hartfelder

**Affiliations:** 1 Cardiosurgery, Republic Cardiology Clinic, Cheboksary, Russian Federation; 2 Medical Faculty, Ulianov Chuvash State University, Cheboksary, Russian Federation; 3 Social And Clinical Psychology, Ulianov Chuvash State University, Cheboksary, Russian Federation

**Keywords:** risk factor, time perspective, Depression, cardiac surgery patients

## Abstract

**Introduction:**

Depression is one of common comorbid states that accompany cardiovascular diseases. Risk of co-morbidity can rise when patients have to undergo heart surgery, which is an additional stress-factor.

**Objectives:**

To specify psychological correlations between depressive manifestations in cardiac surgery patients based on the analysis of their time perspective profile.

**Methods:**

Using the Zimbardo Time Perspective Inventory, we examined 60 cardiac surgery inpatients (80% male, mean age 58.25±10.55). We calculated the statistical estimation of the received data based on the comparison with the norm and the correlation analysis.

**Results:**

The research revealed that cardiac surgery patients’ indices significantly exceeded the norm on three out of five scales – Negative-Past (t=4.405; p=.000), Positive-Past (t=3.536; p=.000), and Future (t=5.008; p=.000). We also identified essential correlations between the level of depression and the indices of Negative-Past (r=.390; p=.002) and Positive-Past (r=-.270; p=.037). We distinguished a positive correlation of the negative attitude to the past with cognitive-affective (r=.369; p=.004) and somatic (r=.338; p=.008) manifestations of depression, and a negative correlation with the level of education (r=-.292; p=.024).

**Conclusions:**

The personal time perspective profile in cardiac surgery patients is unbalanced due to a high level of their negative attitude to the past with an optimal level in other time perspectives. The degree of the Negative-Past attitude correlates in the patients with a low level of education and a high risk of depression in all its manifestations. The given correlations should be taken into account when conducting preventive psychological interventions.

**Disclosure:**

No significant relationships.

